# Adjustment for survey non-participation using record linkage and multiple imputation: A validity assessment exercise using the Health 2000 survey

**DOI:** 10.1177/14034948211031383

**Published:** 2021-08-14

**Authors:** Megan A. Mcminn, Pekka Martikainen, Tommi Härkänen, Hanna Tolonen, Joonas Pitkänen, Alastair H. Leyland, Linsay Gray

**Affiliations:** 1 MRC/CSO Social and Public Health Sciences Unit, University of Glasgow, UK; 2 Usher Institute, University of Edinburgh, UK; 3 Population Research Unit, Faculty of Social Sciences, University of Helsinki, Finland; 4 Public Health and Welfare, National Institute for Health and Welfare (THL), Finland

**Keywords:** Health 2000, Finland, non-participation, alcohol consumption, multiple imputation, validation, record linkage

## Abstract

**Aims:**

It is becoming increasingly possible to obtain additional information about health survey participants, though not usually non-participants, via record linkage. We aimed to assess the validity of an assumption underpinning a method developed to mitigate non-participation bias. We use a survey in Finland where it is possible to link both participants and non-participants to administrative registers. Survey-derived alcohol consumption is used as the exemplar outcome.

**Methods:**

Data on participants (85.5%) and true non-participants of the Finnish Health 2000 survey (invited survey sample *N*=7167 aged 30-79 years) and a contemporaneous register-based population sample (*N*=496,079) were individually linked to alcohol-related hospitalisation and death records. Applying the methodology to create synthetic observations on non-participants, we created ‘inferred samples’ (participants and inferred non-participants). Relative differences (RDs) between the inferred sample and the invited survey sample were estimated overall and by education. Five per cent limits were used to define acceptable RDs.

**Results:**

Average weekly consumption estimates for men were 129 g and 131 g of alcohol in inferred and invited survey samples, respectively (RD –1.6%; 95% confidence interval (CI) –2.2 to –0.04%) and 35 g for women in both samples (RD –1.1%; 95% CI –2.4 to –0.8%). Estimates for men with secondary levels of education had the greatest RD (–2.4%; 95% CI –3.7 to –1.1%).

**Conclusions:**

The sufficiently small RDs between inferred and invited survey samples support the assumption validity and use of our methodology for adjusting for non-participation. However, the presence of some significant differences means caution is required.

## Introduction

Estimates of the prevalence of various health-related behaviours and statuses in a population, typically derived from health surveys, are important for policy and service provision. Whilst designed to be representative of the target population, national health surveys have experienced declining participation levels over recent decades, resulting in smaller and potentially less reliable samples [1]. It is becoming increasingly possible to obtain additional information on health survey participants through record linkage, provided consent is given, in a number of countries where the population register can be used for statistical purposes [2]. Although non-participation does not necessarily result in bias [3], comparisons often find participants alone are not representative of the target population [4,5]. Comparisons of participants and non-participants, in settings where the latter are able to be identified, have revealed differences in baseline socio-demographic characteristics [6] and health outcomes [7–9].

We have previously developed an advanced methodology which aims to mitigate the effect of non-participation bias in health surveys through record linkage to administrative data sources [10,11]. This methodology has been applied in Scotland [12]. It aims to infer observations on non-participants (see terminology in Box 1) using data on the invited survey participants and the population to simulate partial observations on non-participants, which can then be completed through multiple imputation, assuming both Missing At Random and Missing Not At Random scenarios, with the latter offering benefits over conventional weighting approaches. The reliability of this approach depends on the success of the simulation of the inferred non-participants observations. However, the validity of the assumption that the inferred non-participants are representative of the true non-participants is uncertain. Here, we aim to validate this assumption using survey-derived alcohol consumption as the exemplar. The measurement and monitoring of alcohol consumption within a population is of increasing importance, given that it is implicated in a high burden of morbidity and mortality worldwide [13]. The validation exercise requires a health survey setting whereby some information on the true non-participants is known in order to compare to the synthetic observations on non-participants inferred by our methodology. Finland is an ideal setting for such an exercise, as it maintains a nationally representative population register which forms the sampling frame for health surveys and has the ability to record link socio-demographic information, morbidity and mortality databases and survey responses at the individual level using personal identification codes [14]. In particular, socio-demographic characteristics and the occurrence of alcohol-related hospitalisations and all deaths among both the participants and true non-participants are known, and the invited survey sample as a whole can act as a gold-standard comparator. Such population registers are not widely available beyond the Nordic countries, Belgium and the Netherlands [2], and so our validated methodology will provide the opportunity for valuable corrections to survey-derived estimates in many settings.

**Box 1.** Terminology used in this paper.

**Terminology** *Population sample*: An 11% sample of the contemporaneous Finnish population, who were alive and aged between 30 and 79 years on 20 October 2000. *Full invited survey sample*: The original sample of individuals selected to take part in the Health 2000 survey. This comprises those who did participate in the survey, and those who did not (due to refusal, inability to contact, death, etc.) *Invited survey sample*: The full invited survey sample, restricted to those aged 30 to 79 years at baseline. *Participants*: The individuals invited to take part in the Health 2000 survey who were aged 30 to 79 years at baseline, and subsequently participated including returning the questionnaire containing the alcohol consumption questions. *True non-participants*: The individuals invited to take part in the Health 2000 survey who were aged 30 to 79 years at the time of their invitation (established through linkage to the Finnish population register), but did not return the questionnaire containing the alcohol consumption questions. *Inferred non-participants*: The synthetic observations on non-participants, generated through our methodology. *Inferred survey sample*: Comprises the participants and the inferred non-participants, as defined above. *Alcohol-related harms*: either alcohol-related hospitalisations or alcohol-related deaths which occurred during the follow-up period.

## Methods

### Data

**Figure 1.**
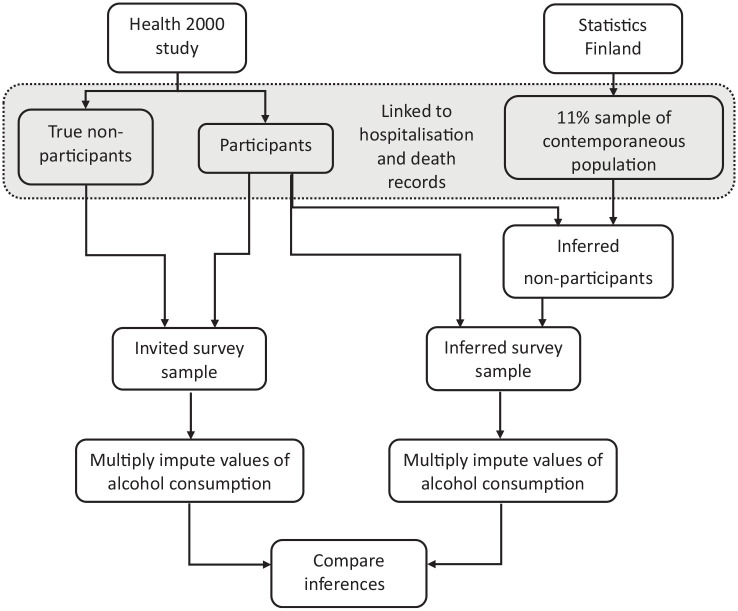
Visual representation of the data used and subsequently generated through this validation process.

To apply the methodology, socio-economic and health data relevant to the survey outcome of interest on the participants and contemporaneous population are required (individually linked for the participant data). For the validation process, we additionally require data on the true non-participants of the invited survey sample, that is, those who were invited to participate but refused. Figure 1 describes the data used and subsequently generated through this validation process.

#### Invited survey sample data

The Finnish Health 2000 survey (thl.fi/health2000) used two-stage cluster sampling to identify 8028 persons aged at least 30 years in 2000 from the individual-level population register in order to create a representative sample of the Finnish population [15]. For this validation exercise, the invited survey sample was restricted to those aged 30–79 years at baseline (*N*=7167) due to the use of oversampling in those aged >80 and evidence of changes to the patterns of alcohol consumption at older ages [16]. The sample sizes for each stratum were proportional to the population size. Thus, the sample was self-weighted and represents the population without weighting in the age group 30–79 years. Post-stratification weights to correct for non-participation were derived by the Health 2000 survey project team for all participants, including those who had participated in other areas of data collection. Weights were based on broad age, sex and local district indicators, as well as design effects (see Appendix). These weights were retained for the application of our methodology but not for the analysis of the total invited survey sample.

The measures of interest for this analysis were collected via questionnaire and included drinking status (current/non-drinker) and average weekly alcohol consumption (grams per week, 1 unit=12 g (Finland)) [17]. We restricted the definition of participants to those who had returned this questionnaire (*n*=6127; 85.5%), and true non-participants were those who had not returned it (*n*=1040), regardless of their participation in any other part of the survey.

#### Population sample data

Analyses of population data in Finland are restricted to an 11% sample of the population aged ⩾15 years permanently residing in Finland at the end of any years in 1987 to 2007. Aggregate counts of those aged 30–79 years and alive on 20 October 2000 (median baseline date for Health 2000; *N*=496,079) were constructed.

#### Educational attainment

Highest level of education attained was available from Statistics Finland’s Register of Completed Education and Degrees [18] and individually linked to the survey and population samples, categorised as primary, secondary and tertiary levels (see Appendix).

#### Linked health outcomes

Records of alcohol-related inpatient hospitalisations and deaths from any cause were individually linked to the invited survey sample and the population sample (1996–2012, obtained from the Finnish Institute for Health and Welfare (THL, hospitalisations [19]) and Statistics Finland (deaths))). Alcohol-related hospitalisations and deaths (harm) were identified using the main diagnosis and additional symptoms (coded using ICD-10; see Appendix). Informed consent for the record linkage was obtained from the Health 2000 participants. Consent was not required for the non-participants and the population sample, as their data were sourced only from register data and used for statistical and scientific purposes [14]. Therefore, hospitalisation and mortality records for the participants, true non-participants and the population sample were available for analysis. Occurrences of alcohol-related admissions prior to the survey participation date (or date of invitation for the non-participants) or 20 October 2000 for the population were excluded.

### Statistical methodology

#### Creating synthetic observations on non-participants: obtaining the inferred survey sample estimates

The methodology used to correct for non-participation bias is described in greater detail elsewhere [11] and in the Appendix. First, we assumed that the expected distributions of age, sex, educational attainment and occurrences of harm and deaths in the invited survey sample were equal to that of the population sample. This is based on the assumption that both samples were representative of the population [11]. Therefore, any deviations in the simultaneous socio-demographic–harm comparisons between the weighted participants of the survey and the population sample elucidated the characteristics of those missing (the ‘non-participants’). Seventy sets of non-participant synthetic observations were inferred based on the deviations in the socio-demographic–harm comparisons, allowing for sampling variation, and combined with the participant observations (total inferred weighted sample sizes ranged between 8006.3 and 8042.3). The resultant inferred survey sample data sets, combining the inferred non-participants and the observed participants, were individual level, containing variables on participation status, age, sex, educational attainment and indicators of whether they had experienced alcohol-related harm, death from any cause, both or neither during follow-up. From the survey, self-reported drinking status and alcohol consumption were available for the participants, and regression-based multiple imputation was used to impute these for the inferred non-participants, as per our methodology [11]. The imputation model contained age and indicators of alcohol-related harm and all-cause mortality, assumed a Missing At Random (MAR) [20] scenario and was stratified by groups of sex and educational attainment, as consumption can vary by socio-demographic status [12]. The results of the imputed data sets were combined using Rubin’s Rules [21] to obtain the inferred survey sample estimates.

#### Imputing alcohol consumption of the true non-participants: obtaining the invited survey sample estimates

The invited survey sample consisted of participants and true non-participants of the Health 2000 survey aged 30–79 (*N*=7167), and used register data on their known socio-demographic variables and known indicators of experiences of alcohol-related harm and all-cause deaths through linkage to the population register and administrative health records. This linkage allowed the invited survey sample to act as a benchmark for comparison to the inferred survey sample, the creation of which was informed by the record-linked data on the participants and register data on the population sample but not by any data on the true non-participants, as above. The same multiple imputation models previously described were applied to the true non-participants’ alcohol consumption and combined as before to obtain the invited survey sample estimates.

#### Validation assessment

In order to assess our methodology’s validity, we followed the approach set out previously [22]. We (a) explored how well the creation and inclusion of the inferred non-participant observations aligned the inferred sample with the population sample; and (b) compared the estimated weekly alcohol consumption of the inferred survey sample to that of the invited survey sample. Each comparison was explored in terms of sex and educational attainment breakdowns. The comparison made in (a) was necessary to check that a sufficient number of inferred samples had been generated and the random rounding used to induce sampling variation was successful, whilst the comparison detailed in (b) enabled an assessment of how well the application of the developed methodology yielded results in line with the invited survey sample if all invited participants had agreed to participate. Differences in each comparison were assessed using the percentage relative difference in mean weekly alcohol consumption estimates, calculated as the difference in alcohol consumption estimates divided by the invited survey sample’s estimate. Relative differences were calculated overall, by sex and by sex and educational attainment. An acceptability limit of ±5% was used to assess the overall validity of the method, with 95% confidence intervals (CI) generated through bootstrap sampling, allowing determination of the statistical significance of any differences [22].

#### Sensitivity analysis

The MAR assumption was relaxed to allow for the possibility that the non-participants are Missing Not At Random (MNAR)—that is, that their non-participation was dependent on their alcohol consumption habits, based on the assumption that non-participation would be associated with higher consumption. Therefore, we allowed for higher consumption in the subgroup of non-participants who experienced harm relative to participants who had also experienced harm. A pattern-mixture approach was used [11] with a hypothesised mean upper limit of 1200 g of alcohol per week for non-participants. This limit was derived through the extrapolation of evidence used in an earlier application of this methodology [23]. This resulted in a series of simulations which explored the impact of the alcohol consumption of non-participants who had experienced harm being re-scaled up to 17 times the sex-specific mean weekly alcohol consumption of participants who had experienced harm. Further details are available in the Appendix. This approach resulted in an adjusted mean consumption of 1189 g and 1152 g among those who had experienced harm in the inferred and invited survey samples, respectively, compared to the MAR estimates of 252 g and 258 g. All analyses were performed using Stata v14.0 (StataCorp, College Station, TX) and the user-contributed ice package [24].

## Results

### Comparison of overall proportions, alcohol-related harm and mortality in each sample

Comparisons of the sex-by-educational attainment compositions of the population, invited survey and inferred survey samples are given in Table I. Whilst the proportions between the weighted participants and the population sample are not vastly different overall, there are indications of the weighting alone not sufficiently adjusting within levels of educational attainment: there is a lower proportion of male participants with primary education (17.7% vs. 18.6% in the population) and a corresponding increased representation of men with tertiary education (13.3%) compared to the population (12.5%). Women with tertiary education are similarly overrepresented in the participants (15.5% vs. 14.7%).

**Table I.** Breakdown of overall proportions, alcohol-related harm and mortality (95% CI) by sex and educational attainment for the sample of the Finnish general population, participants, inferred non-participants, the inferred total, true non-participants and the invited survey sample of the Health 2000 survey.

Level of education	Population sample	Participants^a^	True non-participants	Invited survey sample^b^	Inferred non-participants	Inferred survey sample^c^
*Overall proportions*						
Men						
Primary	18.6	17.7	24.5	18.1	22.8	18.7
	(18.5–18.8)	(16.8–18.7)	(22.0–27.2)	(17.2–19.0)	(18.6–26.9)	(17.6–19.9)
Secondary	17.4	17.3	19.2	17.0	17.5	17.3
	(17.3–17.5)	(16.3–18.2)	(16.9–21.7)	(16.1–17.9)	(13.7–21.3)	(16.2–18.4)
Tertiary	12.5	13.3	11.0	12.5	9.6	12.5
	(12.4–12.6)	(12.4–14.2)	(9.2–13.0)	(11.7–13.3)	(6.5–12.7)	(11.6–13.5)
*All levels*	*48.6*	*48.3*	*54.7*	*47.6*	*49.9*	*48.6*
	*(48.4–48.8)*	*(47.0–49.5)*	*(51.7–57.7)*	*(46.4–48.7)*	*(44.6–55.2)*	*(47.1–50.0)*
Women						
Primary	19.9	19.2	21.1	19.6	22.0	19.8
	(19.7–20.0)	(18.3–20.2)	(18.7–23.6)	(18.7–20.6)	(17.4–26.6)	(18.6–21.0)
Secondary	16.9	17.1	12.7	17.1	16.2	16.9
	(16.8–17.0)	(16.2–18.0)	(10.8–14.9)	(16.3–18)	(11.7–20.6)	(15.7–18.0)
Tertiary	14.7	15.5	11.5	15.6	11.9	14.7
	(14.5–14.8)	(14.6–16.4)	(9.7–13.6)	(14.8–16.5)	(7.7–16.1)	(13.7–15.8)
*All levels*	*51.4*	*51.7*	*45.3*	*52.4*	*50.1*	*51.4*
	*(51.2–51.6)*	*(50.5–53.0)*	*(42.3–48.3)*	*(51.3–53.6)*	*(44.8–55.4)*	*(50.0–52.9)*
*Alcohol-related harm proportions*						
Overall	3.6	3.4	6.2	3.8	4.4	3.6
	(3.6–3.7)	(3.0–3.9)	(4.7–7.6)	(3.3–4.2)	(2.4–6.3)	(3.1–4.2)
Men						
Primary	6.7	6.8	7.8	7.1	6.5	6.8
	(6.6–6.8)	(5.3–8.4)	(4.5–11.2)	(5.7–8.5)	(1.9–11.1)	(5.2–8.4)
Secondary	6.3	5.9	11.0	6.7	6.5	6.1
	(6.3–6.4)	(4.5–7.4)	(6.6–15.4)	(5.3–8.2)	(0.0–13.3)	(4.3–7.9)
Tertiary	3.0	3.5	3.5	3.5	2.1	3.3
	(2.9–3.0)	(2.2–4.8)	(0.1–6.9)	(2.3–4.7)	(0.0–6.3)	(2.0–4.6)
*All levels*	*5.6*	*5.6*	*8.1*	*6.0*	*5.7*	*5.6*
	*(5.5–5.7)*	*(4.8–6.4)*	*(5.8–10.3)*	*(5.2–6.8)*	*(2.3–9.1)*	*(4.7–6.6)*
Women						
Primary	2.2	1.8	3.2	2.0	3.6	2.2
	(2.2–2.3)	(1.0–2.5)	(0.9–5.5)	(1.3–2.7)	(0.0–7.1)	(1.2–3.1)
Secondary	1.8	1.8	6.1	2.3	2.1	1.8
	(1.8–1.9)	(1.0–2.5)	(2.0–10.2)	(1.4–3.1)	(0.0–5.2)	(1.0–2.7)
Tertiary	1.0	0.6	2.5	0.8	3.5	1.1
	(1.0–1.1)	(0.1–1.1)	(0.0–5.3)	(0.3–1.3)	(0.0–7.8)	(0.3–1.8)
*All levels*	*1.7*	*1.4*	*3.8*	*1.7*	*3.0*	*1.7*
	*(1.7–1.8)*	*(1.0–1.8)*	*(2.1–5.6)*	*(1.3–2.1)*	*(0.7–5.4)*	*(1.2–2.3)*
*All-cause mortality proportions*						
Overall	13.7	13.1	18.8	13.6	16.4	13.7
	(13.6–13.9)	(12.2–13.9)	(16.5–21.2)	(12.8–14.4)	(12.4–20.4)	(12.7–14.8)
Men						
Primary	26.8	26.4	28.6	26.7	28.2	26.8
	(26.6–26.9)	(23.7–29.1)	(23.1–34.2)	(24.3–29.1)	(18.6–37.9)	(23.7–29.9)
Secondary	10.4	9.8	8.5	9.6	11.3	10.1
	(10.3–10.5)	(8.0–11.7)	(4.6–12.4)	(8.0–11.3)	(4.5–18.0)	(8.1–12.1)
Tertiary	8.6	9.3	10.5	9.4	6.8	8.9
	(8.5–8.7)	(7.2–11.3)	(4.9–16.2)	(7.5–11.3)	(0.0–16.2)	(6.7–11.1)
*All levels*	*16.2*	*15.8*	*17.9*	*16.1*	*18.1*	*16.2*
	*(16.1–16.3)*	*(14.4–17.1)*	*(14.8–21.1)*	*(14.8–17.3)*	*(12.7–23.5)*	*(14.7–17.8)*
*Women*						
Primary	20.6	19.7	33.8	21.4	23.5	20.6
	(20.4–20.7)	(17.4–22)	(27.5–40.1)	(19.3–23.6)	(13.7–33.3)	(17.8–23.3)
Secondary	6.8	5.9	9.8	6.1	10.2	6.7
	(6.7–6.9)	(4.5–7.3)	(4.7–15)	(4.8–7.4)	(3.8–16.6)	(5.0–8.4)
Tertiary	4.3	4.4	5.8	4.4	4.9	4.5
	(4.3–4.4)	(3.1–5.7)	(1.6–10.0)	(3.2–5.6)	(0.0–11.5)	(3.0–6.0)
*All levels*	*11.4*	*10.6*	*20.0*	*11.3*	*14.8*	*11.4*
	*(11.3–11.5)*	*(9.5–11.7)*	*(16.3–23.6)*	*(10.3–12.3)*	*(9.4–20.1)*	*(10.0–12.7)*

All aged 30–79 years.

a Incorporates sampling weights calculated in the Health 2000 survey.

b Participants and true non-participants combined; no survey weights are incorporated.

c Participants and inferred non-participants combined; participants are weighted, and all inferred non-participants have the null weight value of 1.0.

CI: confidence interval.

Comparison of the composition of the inferred survey sample and the population sample reveal that the appropriate balance has been achieved through the creation of the synthetic observations on non-participants. All health outcome comparisons of the inferred survey sample and the population sample reveal a ⩽0.3% absolute difference.

Relative to the invited survey sample, there was a higher absolute proportion of men in the inferred survey sample (+1.0%), with the greatest difference found in those with primary education (+0.6%); there was a lower proportion of women in the inferred survey sample (–1.0%), with the greatest absolute difference concentrated in those educated to a tertiary level (–0.9%). In terms of health outcomes, a greater absolute proportion of men and women with secondary levels of education experienced alcohol-related harm in the invited survey sample compared to the inferred (men: +0.6%; women: +0.5%), whilst lower absolute proportions of the invited survey sample died during follow-up (men: –0.5%; women: –0.6%).

### Comparison of weekly alcohol consumption estimates in the inferred and invited survey samples

Overall, weekly alcohol consumption is estimated in both samples to be approximately 80 g per week, with a relative difference of just –0.3% (95% CI –1.0% to 0.5%) between the inferred and invited survey samples (Table II), indicating that the creation of the non-participants and their subsequent imputation were successful overall. By sex, the inferred survey sample underestimated the male weekly consumption by 1.6% (95% CI –2.2% to –0.04%), whilst the relative difference for female estimates was –1.1% (95% CI –2.4% to –0.8%). All relative differences remained within the 5% acceptability limit.

**Table II.** MAR imputed estimates of alcohol consumption (g/week) in the inferred and true Health 2000 survey samples for those aged 30–79 years by sex and educational attainment.

	Inferred total sample^a^		Invited survey sample^b^		Relative difference (%)	95% CI
	Mean (g)	95% CI	Mean (g)	95% CI		
Overall	80.1	75.2–85.0	80.4	76.4–84.3	–0.3	–1.0 to 0.5
Men	128.6	119.6–137.7	130.7	123.2–138.2	–1.6	–2.2 to –0.04
Primary	103.0	90.0–116.0	103.8	92.7–114.8	–0.8	–2.3 to 0.8
Secondary	150.4	133.0–167.9	154.2	139.5–168.8	–2.4	–3.7 to –1.1
Tertiary	136.8	121.4–152.3	137.9	124.9–150.8	–0.7	–2.1 to 0.7
Women	34.3	31.6–37.0	34.7	32.2–37.2	–1.1	–2.4 to –0.8
Primary	26.8	22.2–31.5	26.9	22.2–31.5	–0.1	–2.7 to 2.5
Secondary	35.4	30.7–40.0	35.8	31.9–39.6	–1.2	–2.8 to 0.4
Tertiary	43.1	38.4–47.8	43.4	39.1–47.7	–0.6	–2.1 to 0.9

a Participants are weighted; inferred non-participants have a null weight of 1.0.

b No survey weights are incorporated.

MAR: Missing At Random.

### Sensitivity analyses

The results for three MNAR sensitivity analyses are reported in Table III (see Appendix for all MNAR adjustments). Overall weekly alcohol consumption increased from 80 g per week under MAR to approximately 115 g per week under the most extreme MNAR scenario. The relative differences remained within the 5% acceptability limits until it was assumed that non-participants who experienced harm consume up to and including four times the sex-specific mean consumption of the participants.

**Table III.** MNAR imputed estimates of alcohol consumption (g/week) in the inferred and true Health 2000 survey samples for those aged 30–79 years by sex and educational attainment.

	Inferred total sample^a^		Invited survey sample^b^		Relative difference (%)
	Mean	95% CI	Mean	95% CI	
*MNAR2 – 2 times the sex-specific mean weekly alcohol consumption*					
Overall	82.3	77.0–87.5	82.6	78.5–86.6	–0.4
Men	132.4	122.8–142.0	134.8	127.1–142.5	–1.8
Primary	108.3	94.6–122.0	108.5	97.1–119.9	–0.2
Secondary	154.5	135.8–173.3	159.7	144.7–174.7	–3.3
Tertiary	137.9	122.0–153.8	139.2	126.1–152.4	–1.0
Women	34.9	32.2–37.6	35.2	32.7–37.7	–0.7
Primary	27.7	22.9–32.5	27.3	22.7–32.0	1.2
Secondary	35.7	31.0–40.5	36.4	32.5–40.3	–1.8
Tertiary	43.7	38.9–48.4	43.6	39.3–48.0	0.03
*MNAR10 – 10 times the sex-specific mean weekly alcohol consumption*					
Overall	99.4	87.6–111.2	100.4	93.5–107.2	–1.0
Men	162.4	139.3–185.6	168.0	154.4–181.7	–3.3
Primary	150.6	115.5–185.7	146.4	123.9–168.9	2.9
Secondary	187.0	140.8–233.2	204.2	177.8–230.7	–8.4
Tertiary	146.0	120.3–171.6	150.2	131.7–168.7	–2.8
Women	39.8	34.9–44.7	39.0	35.7–42.2	2.1
Primary	34.5	25.7–43.2	31.3	25.5–37.1	10.2
Secondary	38.9	31.6–46.1	41.6	35.8–47.3	–6.4
Tertiary	47.9	40.6–55.3	45.8	40.7–50.8	4.8
*MNAR 17 – 17 times the sex-specific mean weekly alcohol consumption*					
Overall	114.4	95.4–133.4	115.9	105.4–126.4	–1.3
Men	188.7	150.7–226.7	197.1	176.0–218.2	–4.3
Primary	187.7	128.8–246.5	179.5	143.8–215.3	4.5
Secondary	215.5	139.8–291.1	243.2	202.3–284.1	–11.4
Tertiary	153.1	114.9–191.2	159.9	133.8–186	–4.3
Women	44.0	36.3–51.8	42.3	37.9–46.6	4.2
Primary	40.4	26.6–54.2	34.7	27.1–42.3	16.4
Secondary	41.6	31.0–52.2	46.1	37.8–54.4	–9.6
Tertiary	51.7	40.7–62.7	47.6	41.2–54.1	8.6

a Participants are weighted; inferred non-participants have a null weight of 1.0.

b No survey weights are incorporated.

MNAR: Missing Not At Random.

## Discussion

This analysis aimed to validate the assumption of equivalence in simulation of non-participants using a developed methodology which harnesses record linkage and reference population data to create partial observations on non-participants of a health survey and imputes to correct for non-participation bias. We explored differences between the invited and inferred survey samples under both MAR and MNAR assumptions.

The evidence yielded mixed results. The relative differences of the estimates of alcohol consumption for the inferred and invited survey samples were all within our 5% acceptability limits, assuming MAR. However, statistically significant relative differences were estimated for men and women overall and men with secondary levels of education, suggestive of an underestimation from the methodology. Under MNAR, the possibility of the non-participants being systematically heavier drinkers was explored by increasing the amount of alcohol imputed for the non-participants who had experienced harm in both the inferred and invited survey samples. Mean weekly alcohol consumption increased from 80 g overall per week to 115 g in the most extreme scenario. The relative differences between the inferred and invited survey samples increased as the scenarios extremity increased (largest RD=16.4%, women with primary levels of education). Caution is therefore advised in future applications when MNAR assumptions reach their extremities. Results from comparisons of both the inferred sample and the invited sample differed from the results of the weighted participants alone mainly for the MNAR (data not shown).

Broadly speaking, the creation of the observations on non-participants was successful, with the proportion breakdown in the inferred survey sample generally closely reflecting that of the population sample. Men with secondary levels of education were found to consume the largest amounts of alcohol across the sexes and attainment levels in the inferred and invited survey samples and in the participants alone. Table I revealed that higher proportions of the male invited survey sample experienced alcohol-related harm at all levels of educational attainment than would be expected based on the population sample. The greatest difference in rates of incident and any alcohol-related harm between Health 2000 participants and non-participants have previously been found to be within men with secondary levels of education [25].

The strengths of this validation exercise lie within the linked health survey and population sampled data available. Each had 12 years of complete individually linked follow-up data available for participants, non-participants and the population sample. There are several limitations to consider. As alcohol consumption is a survey-derived variable, we do not have observed alcohol consumption for the total sample available for use as a true gold standard. Differences in the proportion breakdown between the invited survey sample and the population sample, especially within those who experienced alcohol-related harm, may indicate violation of the assumption of the survey sample being representative of the population. Third, the relatively high participation rate of the Health 2000 survey (85.5% of those aged 30–79 years) is rare in recent health surveys, with many experiencing response rates <50% [26]. This methodology has been applied to surveys with lower response rates [12], where the inferred sample reflected the population breakdown well but may still require further validation. Finally, this methodology was developed with application to data from Scotland using an area-based measure of deprivation as the measure of socio-economic status. In Finland, no such measure officially exists that is contemporaneous with the survey baseline (the year 2000). Therefore, an individual measure – educational attainment – was used. The elevated risk of alcohol-related harm in lower socio-economic groups has been found to be stronger when measured at the individual level rather than area level [27]. Therefore, this methodology may require further validation for settings with area-level measures.

Alternative approaches to correcting for non-participation include applying calibration weights for MAR or Bayesian modelling [28] and selection modelling [29] for MNAR scenarios. Our methodology offers an advantage over the developed Bayesian approach in that it infers the necessary information on non-participants and can therefore be applied in a wider range of settings, such as those where individual-level register data are not available. The calibration weights and selection modelling approaches will be explored in future work, and they may offer advantages over this methodology, as the technical requirements are more modest. Simulation to compare estimates’ relative finite sample properties will be further explored. Whilst this methodology had previously been used to improve estimates of population-level alcohol consumption [11,12], and this validation exercise continues to make use of alcohol consumption, it could equally be applied to other health-related behaviours of interest, where suitable health records are available and there is a clear link between the health behaviour and harm resulting in hospitalisation or death, such as tobacco smoking.

In conclusion, the validation process presented here indicates that the methodology may be a valid approach to correcting for non-participation bias in health surveys, though consideration of alternatives such as selection modelling is warranted. Especially where lower levels of participation are experienced, the absence of such methodological correction is likely to yield biased results.

## Supplemental Material

sj-docx-1-sjp-10.1177_14034948211031383 – Supplemental material for Adjustment for survey non-participation using record linkage and multiple imputation: A validity assessment exercise using the Health 2000 survey Click here for additional data file. Supplemental material, sj-docx-1-sjp-10.1177_14034948211031383 for Adjustment for survey non-participation using record linkage and multiple imputation: A validity assessment exercise using the Health 2000 survey by Megan A. Mcminn, Pekka Martikainen, Tommi Härkänen, Hanna Tolonen, Joonas Pitkänen, Alastair H. Leyland and Linsay Gray in Scandinavian Journal of Public Health
