# Supplementary material for: Adjustment for survey non-participation using record linkage and multiple imputation: A validity assessment exercise using the Health 2000 survey
Source: Scand J Public Health. 2021 Aug 14;51(2):215–24. doi: 10.1177/14034948211031383 (PMC7614246; doi:10.1177/14034948211031383)
Supplement: sj-docx-1-sjp-10.1177_14034948211031383 – Supplemental material for Adjustment for survey non-participation using record linkage and multiple imputation: A validity assessment exercise using the Health 2000 survey [file sj-docx-1-sjp-10.1177_14034948211031383.docx]

Appendices

Article title: Adjustment for survey non-participation using record-linkage: a validation exercise using the Health 2000 survey

Authors: Megan A. McMinn, Pekka Martikainen, Tommi Härkänen, Hanna Tolonen, Joonas Pitkänen, Alastair H. Leyland, and Linsay Gray

## Further detail on methodology

A full methodology paper describing the process, and Stata syntax are available. ^1^ The methodology requires:

(1) The number of participants (known) and non-participants (unknown, must be estimated);

(2) Aggregate counts of (a) participants and (b) contemporaneous population by age, sex, a measure of socioeconomic position, alcohol-related harms, and deaths experienced during follow-up. The data for the participants are obtained through record-linkage of participants’ data to health registers if consent is provided. Population counts are obtained through administrative data sources.

In order to estimate the number of observations on non-participants to generate in (1), a target sample size of 8,028 was used. This is the total number of people, aged 30 and over, invited to take part in Health 2000. The lower total of 7,126 persons aged 30 to 79 years was not used, as in many settings, the age ranges of the full invited sample are not known, hence the age-restricted sample size would be unknown. Therefore, the participation level used to infer the number of non-participants to be created was 76.3% (6,127 participants out of a potential 8,028).

Combining information from (1) and (2), we are able to infer the number of non-participants within each sociodemographic-harm-death strata by comparing the composition of the strata simultaneously in the *participants* to that of the *population sample*. As the *invited survey sample* was designed to be representative of the Finnish population, deviations between the *weighted* *participants* and the *population* compositions would indicate where there may be unobserved participants (i.e. true non-participants), and therefore where *non-participants* are *inferred.*

This process of inferring non-participants was repeated 70 times, using random rounding, to introduce some sampling variation.

The *inferred survey samples* are created by appending each of the *inferred non-participants* sets generated above onto data on the known *participants*. The resultant 70 data sets had weighted sample sizes ranging between 8,006.3 and 8,042.3, which includes the original Health 2000 sample size of 8,028.

Each dataset is individual level, and contains variables on participation status (*participant* or *inferred non-participant*), age, sex, educational attainment, and indicators of whether they experienced alcohol-related harm, a death from any cause, both, or neither. In addition, the *participants* have observed measurements of alcohol consumption status (current drinker/non-drinker) and the average weekly amount of alcohol consumed. A logistic regression MI model was used to impute the *inferred non-participants’* alcohol consumption status, whilst the amount of alcohol they consumed was imputed using linear regression, on the condition that their imputed alcohol status was “current drinker”. The regression models contained age, and the indicators of alcohol-related harm and all-cause mortality, and were stratified by sex and educational attainment. A sensitivity analyses exploring the effects of including sex and educational attainment in the regression models, rather than being stratifying variables, was run, finding an overall decrease of 1g in the non-stratified models. The largest difference was 2.5g, in men with secondary levels of education (results not shown). A Missing At Random (MAR) scenario is assumed initially, with Missing Not At Random (MNAR) considered in the sensitivity analyses. Imputation was performed within groups of sex and educational attainment, as consumption is known to vary by sociodemographic status. The 70 sets of synthetic observations on non-participants are each imputed once, and the results combined using Rubin’s Rule [ref] to produce a non-participation-corrected mean weekly alcohol consumed estimate for the total sample, by sex, and by measure of socioeconomic position.

Assumptions of the methodology

There are two key assumptions which must be made for the results to be generalisable to the target population. They are discussed in detail in Gray et al. (2019).

Firstly, we assume that the distribution of socidemographic covariates and health outcomes in the population is the same as that of the target survey sample. This assumption can be considered valid if the sampling frame for the survey is representative of the general population.

Secondly, when we impute assuming a Missing At Random scenario, we are explicitly assuming that, conditional on the sociodenomigraphic characteristics and health outcomes, the distribtion of alcohol consumption is independent of participation status. That is, the distribtion of alcohol consumption is the same in the participants and non-participants of the same sex, age group, educational attainment and health status.

Further detail on the MNAR assumptions in the sensitivity analyses

The second assumption is relaxed, and the distrubtion of alcohol consumption is no longer assumed to be the same in participants and non-participants. We modify the imputation model using a pattern-mixture approach to reflect potential differences in the distribution of alcohol consumption by participation status. We specify a value for the sex-specific mean difference in alcohol consumption between the participants and the non-participants, after adjusting for the observed covariates. Under MAR, this value is zero.

We explore the possibility of the non-participants who expereince alcohol-related harm in the follow-up period being a subgroup of very heavy drinkers. In this validation excercise we assume that this subgroup of very heavy drinkers consume up to 17 times the obseved mean alcohol consumption of the participants who had experienced harm. The development of this methodology was conducted using a series of Scottish health surveys (Gorman et al. 2017), which informed their MNAR exercise using data on patients hospitalised in Scotland with serious alochol problems having a mean consumption of 198 units per week. As the mean weekly alcohol consumption imputed in this analysis was approximately half of what was previously estimated in Scotland, we set the upper limit of the amount of alcohol consumed by non-participants who expereince harm to 100 units (1200g of alcohol in Finland). This results in a set of simulations where the alcohol consumption of non-participants who experienced harm during follow-up being successively scaled up until they consumed approximately 100 units more than non-participants who had experienced harm (approximately 17 successive increases).

Further detail on the *Invited Survey Sample*

A full methodology report for the Health 2000 survey is available. ^2^

The Health 2000 survey collected data from its participants in home interviews, health examinations and three self-completed questionnaires. Responses on alcohol consumption were collected from one of the self-completed questionnaires, and the survey respondents who responsed to this were considered to be the *participants* for our purposes, regardless of whether they had contributed to other sections of the survey.

Post stratification weights were calibrated using multiple variables: a design weight (based on the adjusted inclusion probability due to the multistage sampling procedure), health centre district, university hospital district, age (10-year grouping categories), sex and native language (Finnish or Swedish).

## Further details on Educational attainment

The highest level of education attained by the participants and true non-participants were coded using the Finnish Standard Classification 1997 , and the equivalent 2007 version ^3^ was used for the *population sample*. Educational attainment in both data sources was as recorded on 31 December 2000, except for those in the *population sample* who died between 20 October and 31 December 2000 (<1% of population sample); those individuals were assigned their educational attainment of the previous year.

Table A1: ICD 10 Codes used to identify Alcohol related hospitalisations and deaths

| Diseases and Related Health Problems | ICD 10 |
| --- | --- |
| Alcohol induced Pseudo-Cushing’s syndrome | E24.4 |
| Wernicke’s Encephalopathy | E51.2 |
| Mental and behavioural disorders due to use of alcohol | F10.0 – F10.9 |
| Degeneration of nervous system due to alcohol | G31.2 |
| Special epileptic syndromes - related to alcohol  ^a^ | G40.51 |
| Alcoholic polyneuropathy | G62.1 |
| Alcoholic myopathy | G72.1 |
| Alcoholic cardiomyopathy | I42.6 |
| Alcoholic gastritis | K29.2 |
| Alcohol liver diseases | K70.0 – K70.9 |
| Chronic hepatitis, not elsewhere classified | K73.0 – K73.9 |
| Hepatic fibrosis | K74.0 |
| Hepatic sclerosis | K74.1 |
| Hepatic fibrosis with hepatic sclerosis | K74.2 |
| Other and unspecified cirrhosis of liver | K74.6 |
| Alcohol-induced chronic pancreatitis | K86.0 |
| Maternal care for (suspected) damage to foetus from alcohol | O35.4 |
| Foetus and new-born affected by maternal use of alcohol | P04.3 |
| Fetal alcohol syndrome (dysmorphic) | Q86.0 |
| Finding of alcohol in blood | R78.0 |
| Toxic effect of ethanol | T51.0 |
| Toxic effect of methanol | T51.1 |
| Toxic effect of alcohol; unspecified | T51.9 |
| Accidental poisoning by and exposure to alcohol | X45.0 – X45.9 |
| Intentional self-poisoning by and exposure to alcohol | X65.0 – X65.9 |
| Poisoning by and exposure to alcohol, undetermined intent | Y15.0 – Y15.9 |
| Alcohol deterrents | Y57.3 |
| Evidence of alcohol involvement determined by blood alcohol level | Y90 |
| Evidence of alcohol involvement determined by level intoxication | Y91 |
| Alcohol rehabilitation | Z50.2 |
| Alcohol abuse counselling and surveillance | Z71.4 |
| Alcohol Use | Z72.1 |
| ^a^ Code available in the Finnish adaptation of the ICD-10: Epileptic seizures related to alcohol | |

Table A2: MNAR imputed estimates of alcohol consumption (g/week) in the inferred and selected Health 2000 survey samples for those aged 30-to-79-years by sex and educational attainment

|  | Inferred total sample ^a^ | | Selected survey  Sample ^b^ | | Relative difference (%) |
| --- | --- | --- | --- | --- | --- |
|  | Mean | 95% CI | Mean | 95% CI |  |
| **MNAR 1** *– This is the same as MAR* | | | | | |
| **Overall** | 80.1 | 75.2, 85.0 | 80.4 | 76.4, 84.3 | -0.3 |
| **Men** | 128.6 | 119.6, 137.7 | 130.7 | 123.2, 138.2 | -1.6 |
| Basic | 103 | 89.9, 116.0 | 103.8 | 92.7, 114.8 | -0.8 |
| Secondary | 150.4 | 133.0, 167.9 | 154.2 | 139.5, 168.8 | -2.4 |
| Tertiary | 136.8 | 121.4, 152.3 | 137.9 | 124.9, 150.9 | -0.7 |
| **Women** | 34.3 | 31.6, 37.0 | 34.7 | 32.2, 37.2 | -1.1 |
| Basic | 26.8 | 22.2, 31.5 | 26.9 | 22.2, 31.5 | -0.1 |
| Secondary | 35.4 | 30.7, 40.0 | 35.8 | 31.9, 39.6 | -1.2 |
| Tertiary | 43.1 | 38.4, 47.8 | 43.4 | 39.1, 47.7 | -0.6 |
| **MNAR2** – *2 times the sex-specific mean weekly alcohol consumption* | | | | | |
| **Overall** | 82.3 | 77.0, 87.5 | 82.6 | 78.5, 86.6 | -0.4 |
| **Men** | 132.4 | 122.8, 142.0 | 134.8 | 127.1, 142.5 | -1.8 |
| Basic | 108.3 | 94.6, 122.0 | 108.5 | 97.1, 119.9 | -0.2 |
| Secondary | 154.5 | 135.8, 173.3 | 159.7 | 144.7, 174.7 | -3.3 |
| Tertiary | 137.9 | 122.0, 153.8 | 139.2 | 126.1, 152.4 | -1.0 |
| **Women** | 34.9 | 32.2, 37.6 | 35.2 | 32.7, 37.7 | -0.7 |
| Basic | 27.7 | 22.9, 32.5 | 27.3 | 22.7, 32.0 | 1.2 |
| Secondary | 35.7 | 31.0, 40.5 | 36.4 | 32.5, 40.3 | -1.8 |
| Tertiary | 43.7 | 38.9, 48.4 | 43.6 | 39.3, 48.0 | 0.03 |
| **MNAR3** – *3 times the sex-specific mean weekly alcohol consumption* | | | | | |
| **Overall** | 84.4 | 78.6, 90.2 | 84.8 | 80.5, 89.0 | -0.4 |
| **Men** | 136.1 | 125.5, 146.8 | 139.0 | 130.9, 147.1 | -2.0 |
| Basic | 113.6 | 98.4, 128.7 | 113.2 | 101.1, 125.4 | 0.3 |
| Secondary | 158.6 | 137.6, 179.5 | 165.3 | 149.6, 181.0 | -4.1 |
| Tertiary | 138.9 | 122.3, 155.4 | 140.6 | 127.1, 154.1 | -1.2 |
| **Women** | 35.5 | 32.7, 38.3 | 35.6 | 33.1, 38.2 | -0.3 |
| Basic | 28.5 | 23.5, 33.5 | 27.8 | 23.1, 32.5 | 2.5 |
| Secondary | 36.1 | 31.2, 41.1 | 37.1 | 33.1, 41.1 | -2.5 |
| Tertiary | 44.2 | 39.3, 49.1 | 43.9 | 39.6, 48.3 | 0.6 |
| **MNAR4** – *4 times the sex-specific mean weekly alcohol consumption* | | | | | |
| **Overall** | 86.6 | 80.1, 93.0 | 87.0 | 82.5, 91.5 | -0.5 |
| **Men** | 139.9 | 127.9, 151.9 | 143.1 | 134.5, 151.7 | -2.3 |
| Basic | 118.9 | 101.6, 136.1 | 118.0 | 104.8, 131.1 | 0.8 |
| Secondary | 162.6 | 138.9, 186.4 | 170.9 | 154.1, 187.6 | -4.8 |
| Tertiary | 139.9 | 122.4, 157.3 | 142.0 | 128.1, 155.9 | -1.5 |
| **Women** | 36.1 | 33.1, 39.1 | 36.1 | 33.5, 38.7 | 0.0 |
| Basic | 29.4 | 24.0, 34.7 | 28.3 | 23.5, 33.1 | 3.7 |
| Secondary | 36.5 | 31.4, 41.7 | 37.7 | 33.5, 41.8 | -3.1 |
| Tertiary | 44.7 | 39.6, 49.8 | 44.2 | 39.8, 48.6 | 1.3 |
| **MNAR5** – *5 times the sex-specific mean weekly alcohol consumption* | | | | | |
| **Overall** | 88.7 | 81.5, 95.9 | 89.2 | 84.4, 94.0 | -0.6 |
| **Men** | 143.6 | 130.1, 157.2 | 147.3 | 138.0, 156.5 | -2.5 |
| Basic | 124.2 | 104.4, 143.9 | 122.7 | 108.3, 137.1 | 1.2 |
| Secondary | 166.7 | 139.7, 193.7 | 176.4 | 158.4, 194.4 | -5.5 |
| Tertiary | 140.9 | 122.4, 159.4 | 143.4 | 128.9, 157.8 | -1.7 |
| **Women** | 36.7 | 33.5, 40.0 | 36.6 | 33.9, 39.3 | 0.4 |
| Basic | 30.2 | 24.5, 36.0 | 28.8 | 23.9, 33.7 | 4.9 |
| Secondary | 36.9 | 31.5, 42.3 | 38.3 | 34.0, 42.7 | -3.7 |
| Tertiary | 45.3 | 39.9, 50.6 | 44.4 | 40.0, 48.9 | 1.9 |
| **MNAR6** – *6 times the sex-specific mean weekly alcohol consumption* | | | | | |
| **Overall** | 90.8 | 82.8, 98.9 | 91.5 | 86.3, 96.6 | -0.7 |
| **Men** | 147.4 | 132.1, 162.7 | 151.4 | 141.4, 161.4 | -2.7 |
| Basic | 129.4 | 106.9, 152.0 | 127.4 | 111.6, 143.3 | 1.6 |
| Secondary | 170.8 | 140.2, 201.3 | 182.0 | 162.6, 201.4 | -6.2 |
| Tertiary | 141.9 | 122.2, 161.6 | 144.7 | 129.6, 159.9 | -2.0 |
| **Women** | 37.3 | 33.8, 40.9 | 37.1 | 34.3, 39.8 | 0.8 |
| Basic | 31.1 | 24.8, 37.3 | 29.3 | 24.3, 34.4 | 6.0 |
| Secondary | 37.3 | 31.6, 43.0 | 39.0 | 34.4, 43.5 | -4.3 |
| Tertiary | 45.8 | 40.1, 51.5 | 44.7 | 40.1, 49.3 | 2.5 |
| **MNAR7** – *7 times the sex-specific mean weekly alcohol consumption* | | | | | |
| **Overall** | 93.0 | 84.0, 101.9 | 93.7 | 88.1, 99.2 | -0.8 |
| **Men** | 151.2 | 134.0, 168.3 | 155.6 | 144.8, 166.4 | -2.8 |
| Basic | 134.7 | 109.2, 160.3 | 132.2 | 114.8, 149.5 | 1.9 |
| Secondary | 174.8 | 140.6, 209.1 | 187.5 | 166.5, 208.6 | -6.8 |
| Tertiary | 142.9 | 121.8, 164.0 | 146.1 | 130.2, 162.0 | -2.2 |
| **Women** | 38.0 | 34.1, 41.8 | 37.5 | 34.7, 40.4 | 1.1 |
| Basic | 31.9 | 25.1, 38.8 | 29.8 | 24.6, 35.0 | 7.1 |
| Secondary | 37.7 | 31.6, 43.8 | 39.6 | 34.8, 44.4 | -4.9 |
| Tertiary | 46.3 | 40.3, 52.4 | 45.0 | 40.3, 49.6 | 3.0 |
| **MNAR8** – *8 times the sex-specific mean weekly alcohol consumption* | | | | | |
| **Overall** | 95.1 | 85.2, 105.0 | 95.9 | 89.9, 101.9 | -0.8 |
| **Men** | 154.9 | 135.8, 174.0 | 159.7 | 148.0, 171.4 | -3.0 |
| Basic | 140.0 | 111.4, 168.7 | 136.9 | 117.9, 155.9 | 2.3 |
| Secondary | 178.9 | 140.8, 217.0 | 193.1 | 170.4, 215.9 | -7.4 |
| Tertiary | 143.9 | 121.4, 166.5 | 147.5 | 130.8, 164.2 | -2.4 |
| **Women** | 38.6 | 34.4, 42.7 | 38.0 | 35.0, 41.0 | 1.5 |
| Basic | 32.8 | 25.3, 40.2 | 30.3 | 24.9, 35.7 | 8.2 |
| Secondary | 38.1 | 31.7, 44.5 | 40.3 | 35.2, 45.4 | -5.4 |
| Tertiary | 46.9 | 40.4, 53.3 | 45.2 | 40.4, 50.0 | 3.6 |
| **MNAR9** – *9 times the sex-specific mean weekly alcohol consumption* | | | | | |
| **Overall** | 97.3 | 86.4, 108.1 | 98.1 | 91.7, 104.5 | -0.9 |
| **Men** | 158.7 | 137.6, 179.8 | 163.9 | 151.2, 176.5 | -3.2 |
| Basic | 145.3 | 113.5, 177.2 | 141.7 | 120.9, 162.4 | 2.6 |
| Secondary | 183.0 | 140.8, 225.1 | 198.7 | 174.1, 223.2 | -7.9 |
| Tertiary | 145.0 | 120.9, 169.0 | 148.9 | 131.3, 166.4 | -2.6 |
| **Women** | 39.2 | 34.6, 43.7 | 38.5 | 35.4, 41.6 | 1.8 |
| Basic | 33.6 | 25.5, 41.7 | 30.8 | 25.2, 36.4 | 9.2 |
| Secondary | 38.5 | 31.6, 45.3 | 40.9 | 35.5, 46.3 | -5.9 |
| Tertiary | 47.4 | 40.5, 54.3 | 45.5 | 40.6, 50.4 | 4.2 |
| **MNAR10** – *10 times the sex-specific mean weekly alcohol consumption* | | | | | |
| **Overall** | 99.4 | 87.6, 111.2 | 100.4 | 93.5, 107.2 | -1.0 |
| **Men** | 162.4 | 139.3, 185.6 | 168.0 | 154.4, 181.7 | -3.3 |
| Basic | 150.6 | 115.5, 185.7 | 146.4 | 123.9, 168.9 | 2.9 |
| Secondary | 187.0 | 140.8, 233.2 | 204.2 | 177.8, 230.7 | -8.4 |
| Tertiary | 146.0 | 120.3, 171.6 | 150.2 | 131.7, 168.7 | -2.8 |
| **Women** | 39.8 | 34.9, 44.7 | 39.0 | 35.7, 42.2 | 2.1 |
| Basic | 34.5 | 25.7, 43.2 | 31.3 | 25.5, 37.1 | 10.2 |
| Secondary | 38.9 | 31.6, 46.1 | 41.6 | 35.8, 47.3 | -6.4 |
| Tertiary | 47.9 | 40.6, 55.3 | 45.8 | 40.7, 50.8 | 4.8 |
| **MNAR 11 -** *11 times the sex-specific mean weekly alcohol consumption* | | | | | |
| **Overall** | 101.5 | 88.7, 114.3 | 102.6 | 95.2, 109.9 | -1.0 |
| **Men** | 166.2 | 141.0, 191.4 | 172.2 | 157.5, 186.8 | -3.5 |
| Basic | 155.9 | 117.5, 194.3 | 151.1 | 126.8, 175.4 | 3.2 |
| Secondary | 191.1 | 140.8, 241.4 | 209.8 | 181.4, 238.2 | -8.9 |
| Tertiary | 147.0 | 119.6, 174.3 | 151.6 | 132.1, 171.1 | -3.1 |
| **Women** | 40.4 | 35.1, 45.7 | 39.4 | 36.1, 42.8 | 2.4 |
| Basic | 35.3 | 25.9, 44.8 | 31.8 | 25.7, 37.8 | 11.2 |
| Secondary | 39.3 | 31.6, 47.0 | 42.2 | 36.1, 48.3 | -6.9 |
| Tertiary | 48.5 | 40.6, 56.3 | 46.0 | 40.8, 51.3 | 5.3 |
| **MNAR 12 -** *12 times the sex-specific mean weekly alcohol consumption* | | | | | |
| **Overall** | 103.7 | 89.9, 117.5 | 104.8 | 96.9, 112.7 | -1.1 |
| **Men** | 169.9 | 142.6, 197.2 | 176.3 | 160.6, 192.0 | -3.6 |
| Basic | 161.2 | 119.5, 203.0 | 155.9 | 129.7, 182.0 | 3.4 |
| Secondary | 195.2 | 140.7, 249.6 | 215.4 | 184.9, 245.8 | -9.4 |
| Tertiary | 148.0 | 118.9, 177.1 | 153.0 | 132.5, 173.5 | -3.3 |
| **Women** | 41.0 | 35.3, 46.7 | 39.9 | 36.4, 43.4 | 2.7 |
| Basic | 36.2 | 26.0, 46.3 | 32.3 | 26.0, 38.5 | 12.1 |
| Secondary | 39.7 | 31.5, 47.8 | 42.8 | 36.4, 49.3 | -7.4 |
| Tertiary | 49.0 | 40.7, 57.4 | 46.3 | 40.9, 51.7 | 5.9 |
| **MNAR 13 -** *13 times the sex-specific mean weekly alcohol consumption* | | | | | |
| **Overall** | 105.8 | 91.0, 120.6 | 107.0 | 98.7, 115.4 | -1.1 |
| **Men** | 173.7 | 144.3, 203.1 | 180.5 | 163.7, 197.2 | -3.8 |
| Basic | 166.5 | 121.4, 211.6 | 160.6 | 132.6, 188.6 | 3.7 |
| Secondary | 199.2 | 140.6, 257.9 | 220.9 | 188.5, 253.4 | -9.8 |
| Tertiary | 149.0 | 118.2, 179.8 | 154.4 | 132.8, 175.9 | -3.5 |
| **Women** | 41.6 | 35.5, 47.7 | 40.4 | 36.7, 44.1 | 3.0 |
| Basic | 37.0 | 26.2, 47.9 | 32.8 | 26.2, 39.3 | 13.0 |
| Secondary | 40.1 | 31.4, 48.7 | 43.5 | 36.7, 50.3 | -7.9 |
| Tertiary | 49.5 | 40.7, 58.4 | 46.6 | 40.9, 52.2 | 6.4 |
| **MNAR 14 -** *14 times the sex-specific mean weekly alcohol consumption* | | | | | |
| **Overall** | 108.0 | 92.1, 123.8 | 109.2 | 100.4, 118.1 | -1.2 |
| **Men** | 177.4 | 145.9, 209.0 | 184.6 | 166.8, 202.5 | -3.9 |
| Basic | 171.8 | 123.3, 220.3 | 165.3 | 135.4, 195.3 | 3.9 |
| Secondary | 203.3 | 140.4, 266.1 | 226.5 | 192.0, 261.1 | -10.3 |
| Tertiary | 150.0 | 117.4, 182.6 | 155.7 | 133.1, 178.4 | -3.7 |
| **Women** | 42.2 | 35.7, 48.7 | 40.9 | 37.0, 44.7 | 3.3 |
| Basic | 37.9 | 26.3, 49.5 | 33.2 | 26.5, 40.0 | 13.9 |
| Secondary | 40.5 | 31.3, 49.6 | 44.1 | 37.0, 51.3 | -8.3 |
| Tertiary | 50.1 | 40.7, 59.5 | 46.8 | 41.0, 52.6 | 7.0 |
| **MNAR 15 -** *15 times the sex-specific mean weekly alcohol consumption* | | | | | |
| **Overall** | 110.1 | 93.2, 127.0 | 111.5 | 102.1, 120.9 | -1.2 |
| **Men** | 181.2 | 147.5, 214.9 | 188.8 | 169.9, 207.7 | -4.0 |
| Basic | 177.1 | 125.1, 229.0 | 170.1 | 138.2, 201.9 | 4.1 |
| Secondary | 207.3 | 140.2, 274.4 | 232.1 | 195.4, 268.7 | -10.7 |
| Tertiary | 151.0 | 116.6, 185.5 | 157.1 | 133.3, 180.9 | -3.9 |
| **Women** | 42.8 | 35.9, 49.8 | 41.3 | 37.3, 45.3 | 3.6 |
| Basic | 38.7 | 26.4, 51.0 | 33.7 | 26.7, 40.8 | 14.7 |
| Secondary | 40.8 | 31.2, 50.5 | 44.8 | 37.3, 52.3 | -8.8 |
| Tertiary | 50.6 | 40.7, 60.6 | 47.1 | 41.1, 53.1 | 7.5 |
| **MNAR 16 -** *16 times the sex-specific mean weekly alcohol consumption* | | | | | |
| **Overall** | 112.2 | 94.3, 130.2 | 113.7 | 103.8, 123.6 | -1.3 |
| **Men** | 185.0 | 149.1, 220.8 | 192.9 | 172.9, 213.0 | -4.1 |
| Basic | 182.4 | 127.0, 237.8 | 174.8 | 141.0, 208.6 | 4.3 |
| Secondary | 211.4 | 140.0, 282.8 | 237.6 | 198.8, 276.4 | -11.0 |
| Tertiary | 152.0 | 115.8, 188.3 | 158.5 | 133.6, 183.4 | -4.1 |
| **Women** | 43.4 | 36.1, 50.8 | 41.8 | 37.6, 46.0 | 3.9 |
| Basic | 39.6 | 26.5, 52.6 | 34.2 | 26.9, 41.6 | 15.6 |
| Secondary | 41.2 | 31.1, 51.3 | 45.4 | 37.5, 53.3 | -9.2 |
| Tertiary | 51.2 | 40.7, 61.6 | 47.3 | 41.1, 53.6 | 8.0 |
| **MNAR 17 -** *17 times the sex-specific mean weekly alcohol consumption* | | | | | |
| **Overall** | 114.4 | 95.4, 133.4 | 115.9 | 105.4, 126.4 | -1.3 |
| **Men** | 188.7 | 150.7, 226.7 | 197.1 | 176.0, 218.2 | -4.3 |
| Basic | 187.7 | 128.8, 246.5 | 179.5 | 143.8, 215.3 | 4.5 |
| Secondary | 215.5 | 139.8, 291.1 | 243.2 | 202.3, 284.1 | -11.4 |
| Tertiary | 153.1 | 114.9, 191.2 | 159.9 | 133.8, 186 | -4.3 |
| **Women** | 44.0 | 36.3, 51.8 | 42.3 | 37.9, 46.6 | 4.2 |
| Basic | 40.4 | 26.6, 54.2 | 34.7 | 27.1, 42.3 | 16.4 |
| Secondary | 41.6 | 31.0, 52.2 | 46.1 | 37.8, 54.4 | -9.6 |
| Tertiary | 51.7 | 40.7, 62.7 | 47.6 | 41.2, 54.1 | 8.6 |
| ^a^ Participants are weighted, inferred non-participants have a null weight of 1.0  ^b^ No survey weights are incorporated | | | | | |

1. Gray L, Gorman E, White IR, et al. Correcting for non-participation bias in the health surveys using record-linkage and pattern mixture modelling to multiply impute non-participants *Stat Methods Med Res* 2019.

2. Methodology Report: Health 2000 Survey. Helsinki: KTL-National Public Health Institute, Finland Department of Health and Functional Capacity; 2008.

3. Statistics Finland. Finnish Standard Classification of Education 2007. <https://www.stat.fi/meta/luokitukset/koulutus/001-2007/koko_luokitus_en.html> (accessed 02/11/2018.
